# Validation of the energetics of a Huxley muscle–tendon complex model using experimental data obtained from mouse soleus muscle

**DOI:** 10.1242/jeb.249242

**Published:** 2025-08-01

**Authors:** Koen K. Lemaire, Dinant A. Kistemaker, Richard T. Jaspers, Willem J. van der Laarse, A. J. ‘Knoek’ van Soest

**Affiliations:** ^1^ Department of Human Movement Sciences, VU University Amsterdam, Van Der Boechorststraat 9, 1081 Amsterdam, The Netherlands; ^2^ Laboratory for Myology, Department of Human Movement Sciences, VU University Amsterdam, Van Der Boechorststraat 9, 1081 Amsterdam, The Netherlands; ^3^ Department of Physiology, Amsterdam University Medial Centers, Van der Boechorststraat 7, 1081 BT, Amsterdam, The Netherlands

**Keywords:** Metabolic energy expenditure, Cross-bridge work, Muscle oxygen consumption, Musculoskeletal modeling

## Abstract

The aim of this study was to advance muscle models that unify mechanical behaviour and metabolic energy expenditure. To that end, we compared predictions of force and metabolic energy expenditure of a Huxley-type muscle–tendon complex (MTC) model with previously obtained experimental data. In our published model, we extended the classic Huxley formulation by incorporating force–length dependency, series elasticity and activation dynamics. Metabolic energy expenditure was modelled as the weighted sum of cross-bridge cycling and calcium pumping costs. In the associated experiment, fibre bundles from nine mouse soleus muscles underwent sinusoidal contractions, while oxygen consumption and tendon force were measured. The bundles were stimulated during both shortening and lengthening, and measurements were taken before and after adding blebbistatin, which blocks cross-bridge cycling but leaves calcium handling unaffected. This enabled separate estimation of metabolic energy costs for each process. In the present study, we modelled these previously published data. Parameters governing model mechanical behaviour were calibrated using trials without oxygen measurements. We used these parameters in simulations of the oxygen measurement trials, and metabolic parameters were optimized to best match average metabolic power. We found that simulated and measured forces corresponded well (root-mean-square error, RMSE <10% of maximum force). Metabolic energy predictions showed higher error (mean RMSE 20.3%, s.d. 12.6% of measured value), with large inter-animal variability. In four animals, where repeated measures were consistent and data followed expected trends, predictions of metabolic energy expenditure were accurate (RMSE <15%). In the remaining five, greater variability or inconsistent data patterns led to poorer fits. Despite this, given the within-animal variability in oxygen measurements, the metabolic predictions are promising. Combined with previous findings, these results support the potential of Huxley-type models in predictive simulations of human metabolic energy expenditure.

## Teaser

**Summary:** Evaluation of a Huxley-type muscle–tendon complex model that includes elastic tissues and activation dynamics, using data from mice, shows model predictions are good for the mechanics and promising for the metabolic cost.

## INTRODUCTION

When interpreting important features of the human locomotor system, it is often hypothesized that its design (e.g. Lichtwark and Wilson, 2007; Rodman and McHenry, 1980) and concomitant behaviour (e.g. Selinger et al., 2015) are optimized to minimize metabolic energy expenditure. In addition to direct experimentation, hypotheses regarding the role of metabolic energy expenditure in (the control of) human (loco)motion can be tested with the aid of musculoskeletal models (Koelewijn et al., 2019; van den Bogert et al., 2012; Miller et al., 2012; Umberger, 2010; Kistemaker et al., 2010). Typically, musculoskeletal models employed to test such hypotheses contain Hill-type muscle models to represent the actuators. Owing to the original work by A.V. Hill in 1938, metabolic power is usually modelled as the sum of mechanical power and several heat rate terms, which are related to muscle activation, generation of force and shortening/lengthening of the muscle (Lichtwark and Wilson, 2005; Bhargava et al., 2004; Umberger et al., 2003). In this phenomenological approach, these heat terms are parameterized using data obtained from dedicated experiments aimed at a single heat term. The downside of this approach is that it is unclear whether the results of these isolated experiments are representative of contractions *in vivo*, during which all terms vary simultaneously (Curtin and Barclay, 2023). As such, any number of heat terms may be required to describe a given behaviour. This is a conceptual problem, because the essential feature of muscle tissue is that it can convert metabolic energy into mechanical energy, and that both mechanical behaviour and metabolic energy expenditure result from the same mechanochemical reactions (Smith et al., 2005; Herzog and Ait-Haddou, 2002; Barclay and Curtin, 2023). Incorporating knowledge of the contractile process into the formulation of the muscle model can thus improve the generalizability and structural validity of the model.

The reactions that govern the conversion of metabolic energy into mechanical energy take place during the cross-bridge cycle (Herzog and Ait-Haddou, 2002; Barclay et al., 2010; Huxley, 2000a), and as such cross-bridge cycling determines a large part of the energy required for muscle contraction. In addition to cross-bridge cycling, considerable energy is required in the muscle for the (de)activation process, mainly through the active pumping of intra-cellular Ca^2+^ back into the sarcoplasmic reticulum (Barclay et al., 2007). A mathematical model of the cross-bridge cycling process was first formulated by Huxley (1957). In his scheme, the contractile process is modelled as a time-evolving distribution of the relative fraction of attached cross-bridges over their bond length. In the Huxley model, both muscle force and cross-bridge energy consumption follow from this distribution. In a previous study, we extended the original two-state Huxley model to incorporate parallel and series elasticity, length dependence of contractile element force and activation dynamics, and we showed that this model was capable of capturing the mechanical behaviour of rat soleus muscles during dynamic contractions with constantly varying length and stimulation (Lemaire et al., 2016). Following up on that study, we showed that it is numerically feasible to employ this model in task optimization of a largish-scale musculoskeletal model, and that actuation by muscles represented by Huxley-type cross-bridge models yields mechanical behaviour that is very similar to that obtained using Hill-type muscle models (van Soest et al., 2019). These previous results opened up the possibility of investigating how well metabolic energy expenditure by muscle can be predicted by a Huxley-type muscle–tendon complex (MTC) model. To that end, in an additional study we collected data on the energy consumption of mouse soleus muscle fibre bundles that were subjected to periodic contractions (Lemaire et al., 2019). In that study, the metabolic energy expenditure of the muscle fibre bundles was measured while stimulating the bundles during either the concentric or the eccentric phase. In addition, the metabolic energy expenditure related to cross-bridge cycling was separated from that related to the activation process, by the application of blebbistatin, which blocks the acto-myosin interaction while leaving all other processes intact. In the current study, we investigated the extent to which the previously collected data on muscle mechanical behaviour and metabolic energy expenditure in Lemaire et al. (2019) can be predicted by the Huxley-type MTC model described in Lemaire et al. (2016).

## MATERIALS AND METHODS

### Outline of this study

The Huxley-type MTC model used in the current study was similar to that presented in Lemaire et al. (2016). The experimental data used in this study were previously described in Lemaire et al. (2019). In addition, we used unpublished data that were collected in the same study, consisting of isometric and sinusoidal contractions at various muscle lengths, movement amplitudes and stimulus phases. During these contractions, no oxygen was measured. Parameters governing the mechanical behaviour of the Huxley model were fitted to a subset of the previously collected data, which only concerned measurement of mechanical behaviour. The model predictions of the mechanical behaviour were evaluated by a cross-validation procedure, in which parameters estimated on the data of one animal were used to simulate data from the other animals. Scaling parameters that relate the metabolic energy expenditure associated with cross-bridge cycling and with muscle activation were fitted to the data pertaining to muscle energetics, and this fit was evaluated in terms of its root-mean-square error (RMSE).

### Model formulation

**Fig. 1.**
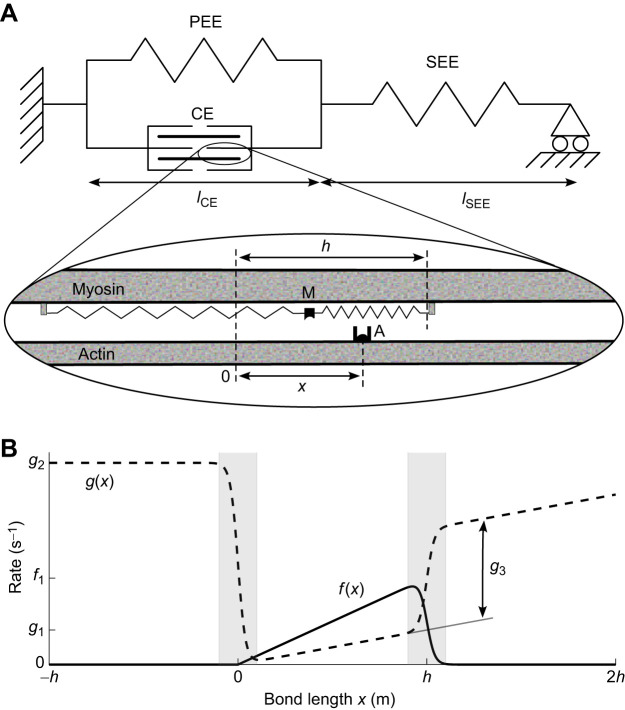
**Macroscopic and microscopic structure of the Huxley-type muscle–tendon complex model and definition of rate functions.** (A) Macroscopic structure of the model. The model consists of a contractile element (CE) and a parallel elastic element (PEE), both in series to a series elastic element (SEE). The expansion depicts the interaction between the myosin and actin filaments that govern the contractile element force production. Cross-bridges form when the M-site on the myosin filament bonds with the A-site on the actin filament. The distance of the A-site to the equilibrium position of the M-site is defined as the bond length *x*, with the maximum distance at which cross-bridges can still form defined as *h*. *l*_CE_ and *l*_SEE_, CE and SEE length. (B) Definition of rate functions for attachment [*f*(*x*), solid line] and detachment [*g*(*x*), dashed line], governing the rate of change of the fraction of attached cross-bridges over time (Eqn 7). The shaded areas indicate the regions over which the original, piecewise linear rate functions were smoothed. The values of *f*_1_ and *g*_1_ on the abscissa refer to *f*(*x*) and *g*(*x*) evaluated at *x*=*h* in the original, non-smoothed functions. For *x*>*h*, parameter *g*_3_ vertically shifts *g*(*x*) compared with the extrapolation of the line *y*=*g*_1_*x*, as indicated by the helper line. Note that in the simulations in the current study, we normalized the bond length *x* by the maximum bond length *h*, and the rate parameters were accordingly scaled.

For a detailed description of the model formulation and derivation of equations, the reader is referred to Lemaire et al. (2016). In brief, the Huxley MTC model consisted of a contractile element (CE) and a parallel elastic element (PEE), which were both aligned in series to a series elastic element (SEE) (Fig. 1A). Assuming muscle mass is small in comparison to the forces it can deliver, the following congruency relations hold:
(1)

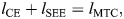

(2)

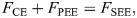
where *l*_CE_, *F*_PEE_ and *F*_SEE_ denoting CE length, PEE force and SEE force, respectively. Both PEE and SEE were modelled as quadratic springs, similar to Lemaire et al. (2016):
(3)

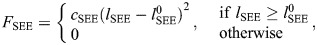
where *c*_SEE_ and 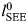 are the series elastic element stiffness parameter and slack length, respectively. The length-dependent stiffness of the SEE equals 2*c*_SEE_*l*_SEE_ and is thus linearly dependent on the stiffness parameter. The stiffness parameter's value was defined by the relative SEE strain at maximum isometric CE force (ε_SEE_), such that 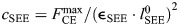 . The equations for the PEE are of exactly the same form. Relative CE isometric force 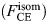 as a function of relative CE length 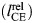, normalized by maximum CE force and optimum CE length, respectively, was modelled as a 4th order polynomial:
(4)

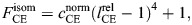
where 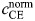 is the normalized CE force–length shape parameter.

Activation dynamics, governing the relationship between muscle stimulation (stim) and the fraction of cross-bridges participating in the contractile process (active state, *q*), was modelled in a similar way to Curtin et al. (1998). Active state, the relative amount of calcium bound to troponin C (Ebashi et al., 1969), was made to depend on the normalized free Ca^2+^ concentration in the sarcoplasmic reticulum (γ), according to a saturating, sigmoid relation:
(5)

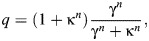
where κ is equal to the value of γ when 50% of the cross-bridges are participating in the contractile process and *n* is the coefficient expressing the cooperativity of binding. The dependency of the free Ca^2+^ concentration on muscle stimulation was governed by first-order dynamics:
(6)

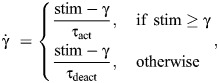
where τ_act_ and τ_deact_ are the activation and deactivation time constants, respectively, and the over-dot represents differentiation with respect to time. As the bundles received only a few stimulus pulses per contraction, we chose to represent each stimulus pulse by a block pulse with a duration of 4 ms and an amplitude of 1. This duration was chosen for numerical convenience, as smaller values would lead to numerically problematic, high-frequency behaviour and larger values would result in undesired fusing at high stimulus frequency. Small deviations in the duration of the stimulus block affected the fitted values of the activation dynamics parameters, but did not affect the overall goodness of the fit of the mechanical behaviour of the model.

The Huxley CE model used in the present study is a modified version of the classic two-state model (Huxley, 1957) in which the time course of the distribution of the fraction of attached cross-bridges (*n*), over their bond length is modelled. In accordance with Huxley's original work, we define the bond length of a (population of) cross-bridge(s) as the distance between the equilibrium position of the myosin head and the nearest actin binding site. Throughout this study, we normalized the bond length by the maximum bond length at which attachment is still possible (*h*), and henceforth will refer to this normalized bond length as *x*. We transformed Huxley's original partial differential equation to a set of ordinary differential equations using the method of characteristics (see Zahalak, 1981), and the input of these differential equations was scaled with both active state and the isometric force–length relationship):
(7)

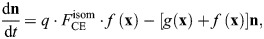

(8)

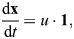
where *f*(*x*) and *g*(*x*) are the attachment and detachment rate functions, respectively, *u* is the velocity of the actin with respect to the myosin filaments and **1** is a vector of size **x** with all entries equal to 1 (vectors are indicated by bold). Following the original work by Huxley (1957), we assume each half sarcomere behaves symmetrically to its counterpart, and the contraction velocity of the sarcomere is twice that of the sliding velocity of the actin filament relative to the myosin filament. Further, assuming that all in-series sarcomeres behave the same, the contraction velocity of the contractile element 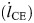 equals that of one sarcomere multiplied by the number of sarcomeres in series *n*_sarc_. Approximating *n*_sarc_ by the optimal contractile element length over the optimal sarcomere length (*s*^opt^), the relationship between filament sliding velocity *u* and CE velocity becomes:
(9)

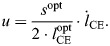
Assuming that each attached cross-bridge exerts a force proportional to its bond length *x*, CE force is proportional to the first-order moment of the distribution *n*(*x*,*t*), according to:
(10)

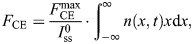
where 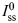 the first-order moment of the steady-state solution of Eqn 7, evaluated at *u*=0, *q*=1 and 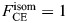. The normalization factor 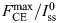 represents the cross-bridge stiffness denoted by ‘*k*’ in Huxley's (1957) original formulation, which we have placed before the integral for clarity.

In our previous study on the mechanics of rat soleus muscle (Lemaire et al., 2016), the shapes of the rate functions *f*(*x*) and *g*(*x*) were chosen to result in a good description of the data whilst being computationally inexpensive. In that study, it was possible to choose rate functions arbitrarily, because a given steady-state force–velocity curve can be adequately fitted with various shapes of rate functions. However, even if the force–velocity relationship is similar for two sets of rate functions, the resulting metabolic energy expenditure may not be similar, because force is related to the number of attached cross-bridges and their bond length, whereas metabolic power is related to the rate of cross-bridge unbinding. In the present study, we chose the shapes for the rate functions to be similar to that originally proposed by Huxley (1957), as these resulted in an adequate description of the energetics of frog muscle. However, the original model was not designed to adequately describe the mechanical behaviour of muscle during eccentric contractions, and has been shown to greatly overestimate the force during eccentric contractions (Zahalak, 1981). Therefore, similar to Zahalak (1981), we added an extra detachment rate parameter *g*_3_, in order to capture the mechanics of eccentric contraction. Furthermore, in the Huxley model, the detachment rate parameter *g*_1_ determines the metabolic energy expenditure during a purely isometric contraction. Because we did not have a separate measurement of muscle metabolic energy expenditure during isometric contraction, we fixed this rate parameter to the arbitrary value of 100 Hz. For numerical reasons, the original rate functions were smoothed by a Gaussian sigmoid over a 0.2*h* wide section at *x*=0 and *x*=h. The resulting rate functions are shown in Fig. 1B. Note that in the current study, we normalized the bond length *x* with the maximum bond length *h*. The values of the rate parameters reported here pertain to this normalized bond length.

Our formulation of the Huxley MTC model has *l*_MTC_(*t*) and stim(*t*) as its inputs, and γ and **n** as its states, with Eqns 6 and 7 as the corresponding state equations. In order to avoid having to numerically find the root of the force balance Eqn 2 at each time step of a simulation, *l*_CE_ was added as a quasi-state variable. As explained in detail in Lemaire et al. (2016), the corresponding state equation can be derived by solving the time derivative of Eqn 2 for 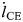.

Total metabolic power was modelled as the sum of metabolic power associated with cross-bridge cycling, *P*_met,cb_, and metabolic power associated with (de)activation, *P*_met,(de)act_. The metabolic energy expenditure associated with all other processes is assumed to be invariant over conditions, and was taken into account by subtracting the resting metabolic energy expenditure, similar to Lemaire et al. (2019). Regarding *P*_met,cb_, we assumed that, in line with Huxley (1957), metabolic power is proportional to the rate of unbinding of cross-bridges 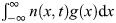. Similarly, as for the mechanical behaviour, the original Huxley model was not designed to capture the metabolic energy expenditure of eccentric contractions. It is well known that, everything else being equal, eccentric contractions require less metabolic energy than concentric contractions (Curtin et al., 2019; Abbott et al., 1952; Fick, 1892). In agreement with these observations, eccentric contraction does require less metabolic energy compared with concentric contraction in the Huxley model, because during eccentric contractions the distribution *n*(*x*,*t*) shifts towards higher bond lengths. This means that for a given force level, concentric contractions involve a larger distribution size than eccentric contractions, and thus have a higher rate of cross-bridge unbinding compared with eccentric contractions. In addition, it has previously been hypothesized that during eccentric contraction, cross-bridges can unbind mechanically, rather than via a chemical reaction involving dissociation of ATP (Loiselle et al., 2010). To model this ‘free’ unbinding, we split the aforementioned integral into two parts, separated at *x*=0.8*h*, and introduced two proportionality constants to scale the rate of cross-bridge unbinding to metabolic power:
(11)

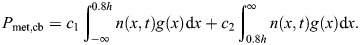
The separation point of the integral was chosen rather arbitrarily, but was found not to affect the current results in terms of the difference between model prediction and experimental data.

To model the metabolic energy expenditure associated with calcium pumping into the sarcoplasmic reticulum, we assumed that (i) the calcium pumps are always operational, (ii) their rate of pumping is linearly dependent on the concentration gradient of calcium between the intracellular space and the calcium concentration in the sarcoplasmic reticulum (γ) and (iii) the amount of metabolic energy expended at the calcium pump is linearly related to the rate of pumping. Under these assumptions, the metabolic power associated with the calcium pumps (*P*_met,act_) is proportional to the free calcium concentration γ:
(12)

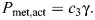
The total metabolic power *P*_met,total_, in watts per kilogram muscle thereby becomes:
(13)

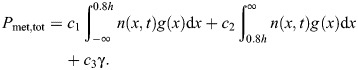
In this equation, *c*_1_ and *c*_2_ have units J kg^−1^ m^−1^ and *c*_3_ has units W kg^−1^.

### Experimental data

The model calibration and evaluation carried out in this study is based on analysis of experimental data on mechanics and energetics of periodic contractions of mouse soleus muscle described in (Lemaire et al., 2019). Below, we will briefly explain the experimental set-up and measurement protocol used in that study.

### Surgery and setup

All experimental procedures were performed in accordance with the regulations of the Animal Experimentation Ethical committee of the Vrije Universiteit Amsterdam (protocol number: DEC FBW1101) and were in accordance with Dutch law. For a complete description of the experimental setup and the experimental conditions, the reader is referred to Lemaire et al. (2019). In brief, 9 C57BL/6J mice were anaesthetized and both soleus muscles were removed from the hindlimbs. A bundle of approximately 70 fibres including their tendon fibres was dissected from one of the muscles and was moved to a jacketed glass chamber containing Tyrode solution equilibrated with a gas mixture containing 95% oxygen and 5% carbon dioxide. The water inside the water jacket was maintained at 32°C. This choice of temperature reflects a trade-off between ecological validity and practical considerations, where we stayed close to the expected physiological temperature, but did not compromise the stability of the preparation. The proximal end of the bundle was attached to the bottom of the chamber. The distal end was attached to a tungsten wire, which left the chamber at the top via a thin capillary and was attached to a servomotor via a force transducer (AE801, SensoNor, Horten, Norway). A custom-made, polarographic oxygen sensor protruded into the chamber through a tightly fitting opening. Through a separate opening, fresh Tyrode solution could be pumped into the chamber. The solution inside the chamber was circulated by a ferritic stainless steel stirrer, which was driven by a magnetic field generated at the bottom of the chamber. The poles of an isolated stimulus generator were attached to stimulus leads placed at the bottom and at the top of the chamber, which were in contact with the Tyrode solution inside. The stimulus isolator generated alternating pulses of 0.4 ms width. Signals from the force transducer, motor position sensor, oxygen electrode and stimulus leads were A/D converted (12 bits) and sampled on a computer at 2 kHz.

### Measurement protocol

For a detailed description of the trials pertaining to measurement of the bundle's metabolic energy expenditure, see Lemaire et al. (2019). In brief, during these trials, the flow of oxygenated Tyrode solution was temporarily stopped, and the bundles were subjected to sinusoidal movements with various amplitudes and frequencies (0.25 mm, 2 Hz; 0.50 mm, 2 Hz; and 0.25 mm, 3 Hz) for a duration of 2 min. Stimulation (5 pulses delivered at 100 Hz) was phased to elicit either concentric or eccentric contraction. For practical reasons, the imposed length changes were in absolute terms. Consequently, the length changes relative to fibre bundle optimum length differed between preparations. Expressed as a percentage of fibre optimal length, the length changes were 4.0±0.5% (range 3.4–4.7%) for the 0.25 mm amplitude condition (see Table 2 for contractile element optimum lengths). After all conditions involving force production were completed, blebbistatin was added to the Tyrode solution. Blebbistatin irreversibly disables the formation of cross-bridges but leaves all other processes unaffected (Kovács et al., 2004; Farman et al., 2008). After addition of blebbistatin, the trials during which oxygen consumption was measured were repeated. The bundle's metabolic energy expenditure in the trials after addition of blebbistatin yielded an estimate of the metabolic energy expenditure that is not associated with cross-bridge cycling. In this study, we assume that this energy expenditure can be ascribed to calcium pumping into the sarcoplasmic reticulum. Between the trials during which oxygen consumption was measured, oxygen content in the chamber was recovered by flushing the chamber with fresh, oxygenated Tyrode solution. During these recovery periods, short-duration contractions were imposed. These included a series of isometric contractions at different bundle lengths, used to characterize the force–length relationship, and a series of isometric contractions at bundle optimum length, with different contraction frequencies (Lemaire et al., 2019). In addition to these isometric contractions, sinusoidal contractions were imposed with various amplitudes (ranging from 0.1 to 0.5 mm), movement frequencies (ranging from 0.5 to 4 Hz) and stimulus trains (ranging from 4 to 8 pulses, always delivered at 100 Hz). Phase of the stimulation with respect to the movement was varied to elicit either concentric or eccentric contraction. Data obtained during the isometric and dynamic trials were used to fit model parameters pertaining to the mechanical behaviour of the Huxley-type MTC model.

### Data analysis

In Lemaire et al. (2016), we performed a series of dedicated experiments aimed at estimating the values of the parameters pertaining to the mechanical behaviour of each model element separately. In Lemaire et al. (2016), it was impossible to perform a similar series of dedicated experiments because of the characteristics of the experimental setup. Instead, we varied the amplitude and frequency of the imposed movement to encompass a wide range of contraction conditions. In the current study, all free parameters pertaining to static properties (i.e. SEE, PEE and CE force–length relationships) were fitted to the isometric trials and all other free parameters were fitted simultaneously to the combined data of all dynamic trials. The values of the parameters pertaining to the energetic behaviour were fitted after the parameter values describing the mechanical part were fitted.

### Parameter fitting

For the (static) force–length relationships of CE, SEE and PEE (Eqns 3 and 4), CE-normalized, SEE strain and PEE slack length were taken to be invariant over animals and their values were based on previous literature reports (see Table 1). Maximum CE force, CE optimum length (constrained to be between 0.9 and 1.1 times the fibre length at rest; based on the force–length curve in Lemaire et al., 2019), SEE slack length and PEE shape parameter were taken to be animal specific and were considered free parameters. For each animal separately, these free parameters were fitted to the data of the force–length relationship by finding the values which minimized the sum of squared differences between the measured and the modelled active and passive force–length relationships, using a constrained optimization method based on gradient descent. For ease of interpretation, the root-mean-squared difference between the modelled and the measured forces is expressed as a percentage of the maximal isometric force. The assumptions regarding the PEE force–length properties of the bundles did not have a large influence on our results as passive forces were of the order of 1% of maximum active force.

**Table 1.** Fixed model parameters shared between animals

Parameter	Symbol	Value
Normalized CE shape	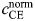	−16^a^
PEE slack length	*n*	0.9^b^. 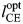
SEE strain at 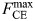	ε_SEE_ (%)	4^c^
Minimum active state	*q*_min_	1e−6^b^
Maximum attachment bond length	*h* (m)	1e−8^d^
Mouse sarcomere optimum length	*s*^opt^ (m)	2.4e−6^a^
Isometric detachment rate parameter	*g*_1_ (Hz)	100^b^

See Materials and Methods for further explanation of variable symbols. ^a^Burkholder and Lieber (2001), ^b^arbitrary value, ^c^van Soest and Bobbert (1993), ^d^Zahalak (1981); Huxley (1957).

Hereafter, for each animal separately, the parameters pertaining to the rate functions of the Huxley model and the time constants of the (de)activation dynamics were fitted simultaneously to both the dynamic contractions and the isometric contractions pertaining to the force–frequency relationship, by finding the set of parameter values which minimizes the following scalar cost function:
(14)

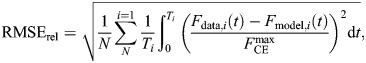
in which *N* equals the number of contractions considered, *T_i_* is the duration of each contraction and *F*_model,*i*_(*t*) is the force resulting from a simulation of the Huxley MTC model with inputs equal to the MTC length and stimulation imposed on the bundle during contraction *i*. Note that we express the relative RMSE (RMSE_rel_) as a percentage throughout, thus multiplied by 100. Similar to the fit of the static force–length relationships, RMSE_rel_ is scaled to have units of force expressed as a percentage of maximum CE force. We minimized RMSE_rel_ through gradient descent optimization, starting from several different randomly chosen points in parameter space. All the optimizations seemed to converge successfully.

The parameter values governing the metabolic energy expenditure (*c*_1_–*c*_3_ in Eqn 13) were estimated for each animal separately, according to the following procedure. For each trial in which metabolic energy expenditure was measured, a simulation was done only of the first contraction in that trial, using the parameter values obtained previously for that animal, and using the bundle length and stimulation as imposed during the experiment. From these simulations, the time-averaged values for the power terms associated with cross-bridge cycling and calcium pumping (see Eqn 13) were obtained. The experimentally observed force in each trial declined throughout the course of the trial. However, the shape of the workloops was similar between subsequent contractions within one trial (see fig. 8 in Lemaire et al., 2019). Because the steady-state value of *n*(*x*,*t*) scales with the relative isometric force, we modelled this within-trial decline in force as a change in the maximum CE force over time, and we scaled the integrals in Eqn 13 with the ratio of the average force obtained during the whole trial to the average force during the first contraction. In the simulations of the trials that were conducted after blebbistatin was added, the integrals in Eqn 11 were set to zero. Equating the modelled *P*_met,tot_ to the measured metabolic power results in one equation with three unknowns *c*_1_–*c*_3_, for each trial. Combining all trials per animal results in an overdetermined system of equations, which is linear in the unknown terms *c*_1_–*c*_3_. First, *c*_3_ was solved separately, by only considering the trials that were conducted after blebbistatin was added. For the remaining set of equations, the now known term *c*_3_·γ was subtracted from the measured metabolic energy expenditure and the remaining parameters *c*_1_ and *c*_2_ were solved in a least squares sense using the Moore–Penrose pseudo inverse.

### Model evaluation

The model was evaluated in terms of its prediction of force by a cross-validation procedure. For each animal, simulations of the data of all other 8 animals were made using the optimal parameter set obtained for this animal, and the difference between these simulation results and the experimental data was taken as a measure of the model's generalizability. For the metabolic energy expenditure, we compared the prediction error with the variation occurring within the experimental data.

## RESULTS

Results are presented as means±s.d. of the sample, with *N*=9, unless mentioned otherwise. All cases in which a typical example is shown or referred to pertain to the same animal for which typical examples were also shown in Lemaire et al. (2019), unless mentioned otherwise.

### Model predictions of mechanical behaviour were good

**Fig. 2.**
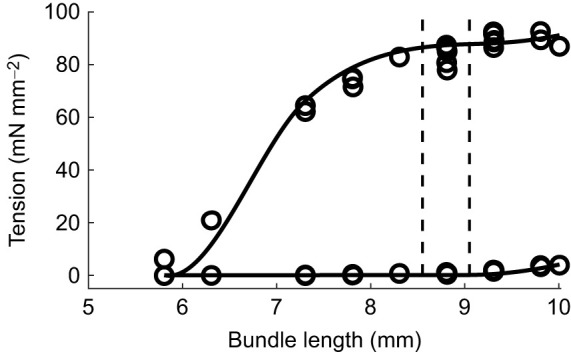
**Typical example of a fitted tension–length relationship.** The open circles are experimental data and the solid line is the model prediction. Vertical dashed lines indicate the length range of the 0.25 mm amplitude condition.

For the fibre bundle of each animal, the force–length relationship could be adequately fitted. The normalized RMSE between the modelled and the measured forces was small (2.3±0.8%), and the fitted parameters were similar for all animals (Table 2). Because of the small between-animal variation in fibre optimum length, we do not expect that the between-animal differences in relative length changes imposed in the dynamic conditions had a substantial effect on other aspects of the model predictions or fits. A typical example of a modelled and measured force–length relationship, for both active and passive conditions, is shown in Fig. 2.

**Table 2.** Fitted model parameters and root-mean-square error (RMSE) pertaining to CE, PEE and SEE force-length relationships

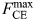 (mN)	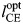 (mm)	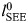 (mm)	ε_SEE_ (%)	RMSE (% 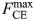 )
16	6.2	2.9	0.52	2.0
12	5.3	2.6	0.16	3.7
14	6.3	2.5	0.16	2.3
24	5.4	2.1	0.86	2.0
12	5.5	2.5	0.34	1.8
18	7.3	2.2	0.39	1.3
19	6.6	2.8	1.00	2.4
22	7.1	2.9	0.36	1.7
19	7.1	2.1	0.64	3.7

Each row in the table refers to the muscle fibre bundle investigated for a single animal. The RMSE refers to the average root-mean-squared difference between the modelled and measured forces, expressed as a percentage of maximal isometric force. See Materials and Methods for explanation of other variable symbols.

**Fig. 3.**
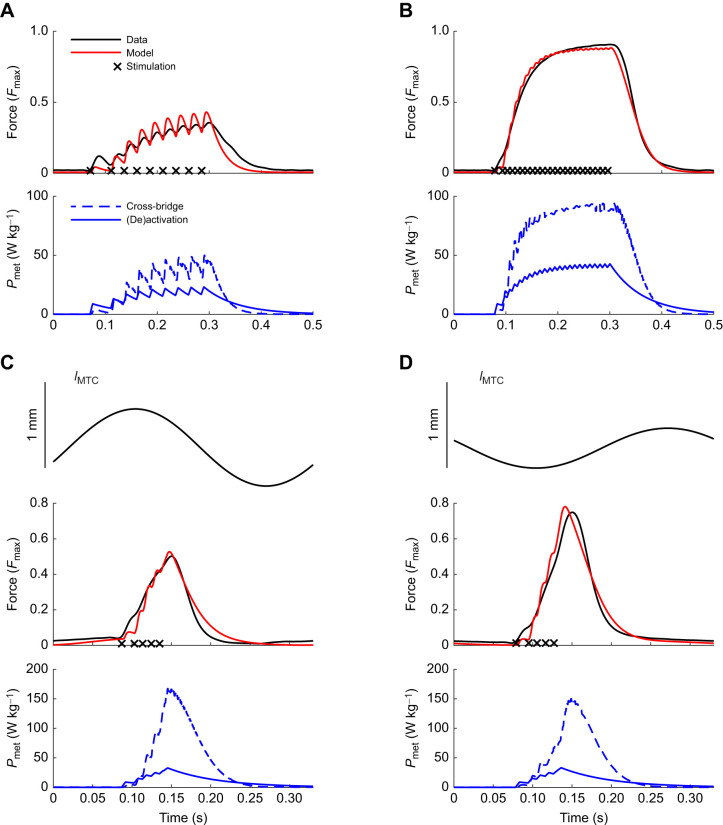
**Experimental force traces and model predictions of force and of metabolic power (typical example).** (A,B) Data of isometric contractions at optimal bundle length, for a low and a high stimulation frequency, respectively. The top graph depicts the measured and modelled normalized force traces, and the bottom graph depicts the modelled metabolic power (*P*_met_) associated with cross-bridge cycling (dashed line) and (de)activation (solid line). (C,D) A concentric and an eccentric contraction, respectively, where the top graph represents muscle length change of the muscle–tendon complex (MTC) with respect to optimum length, with the scale bar representing 1 mm. In all panels, black crosses mark instances of stimulation. Note that for the metabolic power, there are no experimental data shown in this figure as we did not measure metabolic power during these trials. The root-mean-square error (RMSE) values (as calculated from Eqn 14) for the force traces shown in these trials were 2.7%, 4.2%, 3.6% and 4.2% maximum isometric CE force (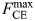) for A–D, respectively.

The dynamic trials could also be adequately described by the model, as evidenced by the small normalized RMSE between modelled and measured forces (5.1±1.6%, Table 3). Typical examples of simulation results for various contraction types and the corresponding data are shown in Fig. 3. We will discuss the modelled metabolic power traces in Fig. 3 in the next section. Note that the RMSE values of the force traces (range 2.7–4.2% of maximal isometric force) are similar to the mean values for all trials listed in Table 3. Given the similarity between modelled and measured force traces in Fig. 3, this indicates good correspondence between modelled and measured forces throughout. Nevertheless, two peculiarities can be noted. First, immediately after the initial stimulus pulse, the predicted forces were lower than the measured values (Fig. 3). This finding was consistent across animals. The experimentally measured force response to stimulation is highly non-linear in this regard; the initial pulse generates a far greater change in force than subsequent pulses. A potential way to incorporate this behaviour in future modelling efforts would be to make the activation dynamics dependent on the recent history regarding stimulation.

**Table 3.** Fitted model parameters and RMSE pertaining to dynamic behaviour

*f*_1_ (Hz)	*g*_2_ (Hz)	*g*_3_ (Hz)	τ_act_ (s)	τ_deact_ (s)	*k*	RMSE (% 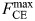 )
3.4e+02	1.6e+04	5.3e+02	0.036	0.079	0.26	4.3
1.4e+03	1.9e+04	3.9e+03	0.090	0.053	0.15	5.5
5.2e+02	7.8e+04	4.8e+02	0.034	0.083	0.40	3.2
7.2e+02	1.3e+04	4.6e+02	0.028	0.070	0.34	6.2
1.2e+03	1.6e+03	1.3e+02	0.022	0.066	0.79	4.8
5.3e+02	1.4e+05	6.2e+02	0.024	0.083	0.41	8.7
7.8e+02	1.1e+04	4.3e+02	0.024	0.075	0.35	5.1
6.4e+02	1.0e+04	4.9e+02	0.033	0.064	0.35	4.1
6.7e+02	1.2e+04	4.0e+02	0.032	0.063	0.40	4.0

Each row in the table corresponds to a single animal. The RMSE in the last column is the value of RMSE_rel_ in Eqn 14 for the dataset of each animal.

The second peculiarity concerns the variation in estimated parameter values between animals. Because the animals have the same genetic background, it might be expected that morphology-independent parameters such as time constants and rate constants would be similar among animals. However, the fitted parameter values differ substantially between animals (Table 3, albeit not by more than one order of magnitude). The time constants in the experimental data are expected to vary with fibre-type distribution, which varied in the experimental data. The bundles had an average of 37±12% type I fibres, ranging from 22% to 53% (Lemaire et al., 2019). Part of the variation in fitted parameter values may thus be related to variations in fibre-type distribution, which were not accounted for in the current model. In future work, introducing a fibre-type distribution dependency of the model's time and rate constants may be expected to reduce the variance in their values. This is desirable, as ideally one (dimensionless) set of parameter values suffices to adequately model the behaviour of each animal.

Contrary to our previous publication (Lemaire et al., 2016), we did not have an independent set of data to validate the mechanical behaviour of the model in the current study. To nonetheless explore the generalizability and to cross-validate the predictions of the mechanical behaviour, we performed simulations of the experimental data pertaining to all animals, using the optimal parameter values fitted to each animal's experimental data. This resulted in 9×9 comparisons of simulated and measured data, for each of which the cost RMSE_rel_ (Eqn 14) was computed. The results of these comparisons in terms of *c* are listed in Table 4. In Table 4, the values in each row pertain to the set of parameter values fitted to the data for a particular animal (corresponding to the parameters listed in each row of Table 3), and the values in each column pertain to simulations of the experimental trials of a particular animal. For example, the value in row 2, column 3 in Table 4 indicates the value of RMSE_rel_ computed from simulations of the data pertaining to animal 3, using parameter values fitted to the data of animal 2. The values on the diagonal are thus identical to the values of RMSE_rel_ listed in Table 3. The cost values in Table 4 can be interpreted as a measure of the quality of the description of the data. The mean normalized RMSE of each set of parameter values of the data of the other 8 animals was 11.4% (s.d. 2.8%). The model predictions are thus reasonably robust to changes in parameter values.

**Table 4.** Model cross-validation

	A1	A2	A3	A4	A5	A6	A7	A8	A9
A1	4.3	9.4	3.5	11.4	16.0	12.4	10.6	6.3	6.9
A2	8.5	5.5	7.4	15.0	14.8	16.6	13.6	5.7	6.5
A3	4.7	8.4	3.2	11.9	14.6	13.6	11.5	5.2	5.7
A4	10.3	13.3	10.0	6.2	22.6	9.0	5.1	10.7	11.7
A5	15.5	14.4	12.4	24.6	4.8	27.1	25.1	14.2	12.0
A6	9.6	13.6	9.5	7.1	22.2	8.7	5.3	10.6	11.6
A7	11.5	14.6	11.3	6.7	23.6	8.9	5.1	12.2	13.1
A8	6.2	6.6	4.6	12.8	13.8	15.0	12.5	4.1	4.2
A9	6.7	6.6	4.9	13.2	13.2	15.7	13.2	4.2	4.0

Values for the cost function RMSE_rel_ (Eqn 14) of the comparison between modelled and measured force, for simulations of each experiment, with each fitted set of parameter values. Values in each row correspond to the same set of parameter values, which are similar to the parameter values listed in Table 3. Values in each column correspond to comparisons with the same set of parameter values. Values on the diagonal are the values for relative RMSE (RMSE_rel_) obtained from each optimization. For example, the value in row 2, column 3, indicates the value of *c* computed from simulations of the data pertaining to animal 3, using parameter values fitted to the data of animal 2.

### Model predictions of metabolic energy expenditure were reasonably good

In Table 5, we show the coefficients *c*_1_–*c*_3_ of Eqn 13; the resulting relative RMSE between the predicted and the measured average metabolic power for the pre-blebbistatin trials; and the relative RSME value for the comparison of predicted and measured forces of these trials. This is shown for each animal in a separate row. As can be appreciated from the last column of Table 5, the relative RMSE of the force predictions is similarly low for these trials as for the data on which the mechanical behaviour was fitted. Moreover, the relative RMSE of the metabolic energy prediction appears to be unrelated to the relative RMSE of the force prediction. Regarding the relative RMSE of the energy predictions of the post-blebbistatin trials (on which only the value of *c*_3_ was fitted, and not listed in Table 5), we note that that is completely determined by the within-animal variation of the empirical data. This is because in the model, we do not distinguish between concentric and eccentric post-blebbistatin trials, as we did not find evidence for such a difference previously (Lemaire et al., 2019). We thus used two post-blebbistatin trials per animal to fit *c*_3_. The mean RMSE of the post-blebbistatin metabolic energy expenditure prediction was 27% (s.d. 14%) of the measured value, or 1.7 W kg^−1^ (s.d. 0.7 W kg^−1^). Thus, the relatively large RMSE reflects the between-trial, within-animal variation, rather than indicating a poor model fit.

**Table 5.** Fitted model parameters and RMSE values pertaining to modelled metabolic energy expenditure

*c*_1_	*c*_2_	*c*_3_	*P*_met,tot_ RMSE	Force RSME (%)
0.16	0.031	159	8.4	4.4
0.16	0.13	340	3.7	5.8
0.19	0.23	93	21.1	8.9
0.10	0.060	56	20.8	6.1
0.38	0.013	48	32.4	10.7
0.18	0.037	48	5.6	6.3
0.14	0.0030	56	9.1	6.5
0.18	0.073	53	13.8	7.3
0.086	0.13	61	41.3	5.7

Each row in the table corresponds to a single animal. Parameters *c*_1_–*c*_3_ pertain to Eqn 13. The *P*_met,tot_ RMSE values are the RMS of the difference between the predicted and the measured metabolic energy expenditure divided by the measured value, for the pre-blebbistatin trials. The force RMSE is the value of Eqn 14, applied to the metabolic trials.

**Fig. 4.**
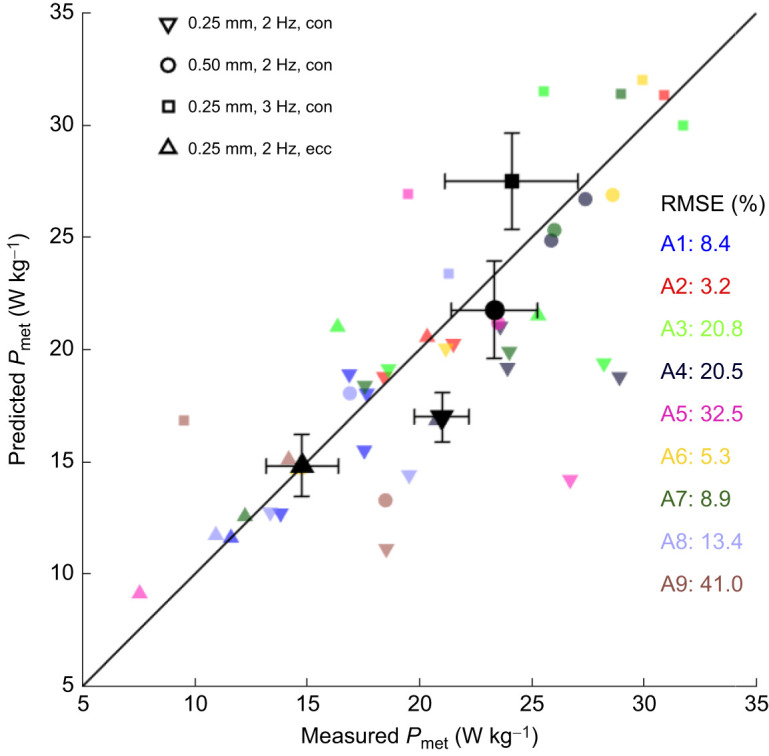
**Scatter plot of measured and predicted metabolic power.** Each small symbol represents one trial, where symbol type indicates the contraction condition (see key for details) and where different animals are distinguished by colour. The larger, black, solid symbols are the mean across animals for each contraction condition. In the case of repeated measures, the within-animal average was used to compute the larger mean. The horizontal error bars represent the variation of the measurement across animals, and the vertical error bars represent the variation in model predictions due to differences in fitted parameter values across animals. The solid black line is the identity line. The coloured numbers are the normalized root mean square (%) of the difference between the predicted and the measured metabolic energy expenditure divided by the measured value. The data in this figure correspond to the normalized RMSE presented in Table 5. con, concentric; ecc, eccentric.

For the pre-blebbistatin trials, the RMSE of the metabolic energy prediction differed substantially between animals (Table 5, column 4). To examine these differences more closely, we plotted the predicted metabolic energy expenditure against the measured values, separated by condition (symbols) and animal (colours; Fig. 4, large symbols indicate the across-animal average per condition, with the error bars indicating across-animal variation of the within-animal means). Note that the average fit for the single eccentric conditions was near perfect, which was expected because there was one free parameter associated with the metabolic energy expenditure during eccentric contractions. Further note that the animals for which the RSME values were large (higher than 15%; animals 3, 4, 5, 8 and 9) had a large within-animal variation in the empirical data. For instance, in animal 5 (pink), both the concentric 0.50 mm, 2 Hz (circle) and the 0.25 mm, 3 Hz (square) condition had a lower metabolic energy expenditure than the concentric 0.25 mm, 2 Hz condition (downward triangle). This result goes against expectations and against the mean trend in the experimental data. In animal 3 (bright green), the empirical results do agree with the mean trend in the data, but there is a large variation (horizontal distance) in repeated measures, which is not reflected by a change in model predictions, as the inputs to the model are the same for repeated trials. In other words, for a given condition and set of parameter values, the model will always yield the same result, whereas the experimental data might differ between successive trials. Any within-animal variation in the empirical trials will therefore directly appear in the RMS difference between model predictions and empirical data. In the animals for which the RMSE between the predicted and measured metabolic energy expenditure was small (lower than 15%; animals 1, 2, 6 and 7), the empirical data more closely followed expected trends (e.g. animal 2, red) and the variation in repeated measures was much smaller (e.g. animal 1, blue). Across animals, the RMSE was 20.3% (12.6%) or 4.3 W kg^−1^ (2.6 W kg^−1^) muscle. To qualitatively assess this prediction, it merits a comparison to the variation, expressed as the RMS difference from the mean value of the trials for which we have repeated measures. These were the 0.25 mm, 2 Hz concentric condition (*N*=7) and the post-blebbistatin (eccentric and concentric) (*N*=9) conditions. The RMS difference values for these conditions were 1.8 W kg^−1^ (s.d. 1.7 W kg^−1^) and 1.2 W kg^−1^ (0.7 W kg^−1^), respectively. This indicates that almost half of the differences between the model predictions and the experimental data might be explained by within-animal variation. In the Supplementary Materials and Methods and Fig. S1, we further investigate the cause of the experimental within-animal variation and conclude that this is at least not due to cell necrosis during the course of the experiment. All in all, given the substantial variation in the experimental data, we consider the model predictions of metabolic energy expenditure to be in reasonable agreement with the experimental results.

To explore the generalizability of these results to other contraction conditions, we simulated some of the trials that were not used in the fitting procedure, and qualitatively assess the simulation results below. In Fig. 3, examples of the predicted metabolic power are shown (bottom graph in each panel), for trials that were not included in the dataset used during parameter fitting of the model of metabolic energy expenditure. Note that no experimental data on the metabolic energy expenditure are shown here, as we did not measure oxygen consumption during these particular trials. Further note that for all contractions, the (de)activation energy expenditure is only dependent on the number and frequency of stimulus pulses. In the isometric contractions (Fig. 3A,B), total metabolic power scales with the force–time integral, as expected. Furthermore, the ratio between (de)activation and total metabolic work was in the expected range, for both low (37%) and high (30%) forces, even though no data from isometric contractions were available for fitting of the parameter values. For the concentric and eccentric contractions, the mechanical efficiency was 0.15 and −0.13, which is similar to previously reported values for mouse soleus muscle (Barclay and Curtin, 2023). These results are by no means conclusive, but they seem at least qualitatively to be in agreement with previous findings, and we thus expect that the predictions of metabolic energy expenditure will generalize well to other contraction conditions. This means that the current modelling effort is suitable for describing the energetics of muscle contraction.

As with the parameters pertaining to the mechanical behaviour, one would expect the parameter values *c*_1_–*c*_3_ to be similar between animals because of their similar genetic background. However, again, there is a fairly large variation in these parameter values (Table 5). This variation is due to both between-animal variation in the empirical data and between-animal variation in the parameters underlying the mechanical behaviour (see above). Similar to the mechanical behaviour, part of the variation in the empirical data may be due to variation in fibre-type distribution. Previously, we found that the percentage of type I fibres was correlated to the mechanical efficiency (Lemaire et al., 2019). Including a fibre-type dependency in the model is therefore expected to reduce the between-animal variation in the values of *c*_1_–*c*_3_. In the current study, we did not do so, because of the risk of over-fitting parameters within one animal. If a fibre-type dependency is added to the current model in future studies, we recommend using a single set of rate constants and muscle activation time constants across animals, where these parameters are scaled by the ratio of type I fibres in such a way that a higher percentage of type I fibres leads to slower dynamics. In this way, fibre-type dependency would affect both the mechanical behaviour and metabolic energy expenditure in an internally consistent manner.

In the general discussion below, we will suggest potential strategies to further improve the prediction of metabolic energy expenditure in this dataset.

## DISCUSSION

The aim of the present study was to investigate how well the data on muscle metabolic energy expenditure presented in Lemaire et al. (2019) can be predicted by the Huxley-type muscle–tendon complex model described in Lemaire et al. (2016). Similar to our previous work, the parameter fitting resulted in good correspondence between predicted and measured forces of the fitted trials. Furthermore, the force predictions generalized well across animals. For the metabolic trials (the data of which were not used to fit the parameters pertaining to mechanical behaviour), the prediction of the force traces was also good. Overall, the prediction of metabolic energy expenditure was reasonably good, even though there were large between- and within-animal variations. Below, we discuss possibilities for improving upon the current study, and future directions.

Similar to Lemaire et al. (2016), we chose to split the parameter-fitting procedure into consecutive stages, as opposed to lumping all free parameters into one large-scale optimization. The reasoning behind this choice was that separate parts of the model can be parameterized in isolation using dedicated experiments. In general, partitioning the parameter estimation procedure is conceptually attractive because it allows us to test and evaluate contained parts of the model separately, and it results in a closer correspondence to observed (muscle) physiology. This may be expected to increase a model's generalizability to conditions other than the ones the parameters were fitted to.

As mentioned before, our method of parameter estimation proved successful in terms of predicting mechanical muscle behaviour. Yet, in contrast to the approach in Lemaire et al. (2016), both the parameters pertaining to the force–velocity relationship (rate parameters) and the parameters governing the activation dynamics were fitted simultaneously in the second stage, as we did not have data from dedicated force–velocity experiments. This introduced the possibility of redundancy in the parameter space, as the activation dynamics and the muscle contractile dynamics constitute two dynamical systems in series, for which different parameter sets can result in similar dynamic behaviour [i.e. similar moments of the distribution *n*(*x*,*t*)]. An example where this may have happened is in animal 2, which had a large value for *f*_1_, combined with a large value for τ_act_ and a low value for κ (row 2 in Table 3). In terms of the overall dynamics of the model, the net effect of these parameter values may well be ‘average’ behaviour. Considering that the large variation in parameter values observed in this study did not lead to large deviations in the quality of the fit (Table 3), it indeed seems that there is redundancy in the parameter space. This redundancy may have had consequences for the prediction of metabolic energy expenditure, which depends on both the activation dynamics and the cross-bridge distribution (Eqn 13). It is thus conceivable that a different combination of rate parameter values and activation dynamics parameter values would have resulted in a least squares solution for the metabolic parameters that has a slightly lower prediction error, while not affecting the description of the mechanical behaviour to any meaningful extent. We chose not to pursue this further in the current study, as we took as a starting point the description of the mechanical behaviour, which can only get worse if an additional term is added to the cost function.

It should be noted that the two-state cross-bridge model as currently presented is a minimal version of the cross-bridge model, which closely follows the original paper by Huxley (1957). For one thing, the contribution of titin to the contractile process is not modelled. Titin is a molecular spring that plays a role predominantly during active eccentric contractions. Through calcium-mediated modulation of its stiffness, titin is hypothesized to explain, amongst others, residual (isometric) force enhancement after stretch (Herzog, 2018). Implementation of the mechanisms in which titin contributes to muscle contraction in a model requires the inclusion of multiple additional states, equations and corresponding parameters (e.g. Millard et al., 2024; Sampaio de Oliveira and Uchida, 2024). Implementing such changes in the current model would most likely improve the predictions of the experimental data, albeit at the cost of introducing additional free parameters and complexity to the model. In the current work, we did not explicitly model the role of myosin-binding protein-C (MyBP-C). MyBP-C is a family of accessory proteins of striated muscles that contributes to the assembly and stabilization of thick filaments, and regulates the formation of actomyosin cross-bridges, via interactions with both the myosin and actin filaments (Ackermann and Kontrogianni-Konstantopoulos, 2011; Ackermann et al., 2015). Three distinct MyBP-C isoforms have been characterized, in cardiac muscle and in slow and fast skeletal muscle. Detailed discussion on how to model the action of MyBP-C in muscle contraction are beyond the scope of this work. However, we do note that any model that incorporates fibre-type dependency in its dynamics would capture some of the effects of different MyBP-C isoforms.

In addition to data from studies on muscle structure, there are behavioural data that were not taken into account in the current modelling effort. There is strong evidence from temperature jump and rapid length change studies that the cross-bridge cycle includes at least one more weakly attached state (for reviews, see Huxley, 2000b; Ranatunga, 2010; Barclay and Curtin, 2023). Including such additional states may improve the overall fit of the data. However, we note that (i) this would again involve adding additional parameters with the associated risk of overfitting and (ii) the additional states were needed to describe the transients due to temperature jumps and rapid length changes; it is unclear whether these are necessary to describe the experiments considered here. Recently, Seow and Seow (2022) suggested that variation in the curvature of the steady-state force–velocity relationship can be interpreted as changes in the velocity dependence of the rate of cross-bridge detachment. In a similar vein, we could consider making the rate of cross-bridge detachment contraction velocity dependent in the current model. Also, we could consider improving upon the model of activation dynamics. Lastly, our assumptions regarding the metabolic energy requirement for the calcium pumps are rather simplistic, and not reflective of the current state of calcium pump models (e.g. Baylor and Hollingworth, 2007; Tran et al., 2009). Incorporating these more realistic models of calcium transport is likely to improve the general validity of the model. In the current study, this may not have had a large effect as the variation in contraction conditions was in terms of contraction velocity and activation phase, and not in terms of activation duration. It would be interesting to investigate the above modifications and additions to the model in future work.

In the above, we argued that different combinations of rate parameter values and activation dynamics parameter values may lead to similar mechanical behaviour but different metabolic energy expenditure. This argument can be extended to different shapes of rate functions, because there is both a many-to-one mapping of distributions *n*(*x*) to force and (a different) many-to-one mapping of distributions *n*(*x*) to metabolic power. Different rate function shapes may thus lead to similar mechanical behaviour but different metabolic power. If so, the latter could be exploited to further reduce the prediction error of the metabolic energy expenditure. As the amount of data for this study is limited, we did not explore these options further, to prevent over fitting.

Finally, given the relatively large variation in the experimental data, it would be helpful if more high-quality data on muscle energetics becomes available. In particular, data from experiments on mammalian muscle at the appropriate temperature and in which the contributions of calcium pumping and cross-bridge cycling to metabolic energy expenditure are separated would be valuable. Such data should be combined with available knowledge on the details of the muscle contraction process, to further develop and validate structural models of the mechanics and energetics of muscle contraction.

### Concluding remarks

As far as the authors are aware, this study is the first attempt to evaluate a Huxley-type MTC model in terms of its simultaneous predictions of mechanical behaviour and metabolic energy expenditure. In our view, the current results are promising; directions for improvement of the analysis and model in future work are provided in the above. In combination with our previous work on the feasibility of implementing a Huxley-type MTC model in musculoskeletal modelling (van Soest et al., 2019), this study represents an important step towards better prediction of whole-body energy expenditure, using the Huxley cross-bridge model. If this proves successful, this will aid investigations into the role of energy expenditure in human motor control, and the design of assistive devices in sports and health applications.

## Supplementary Material

10.1242/jexbio.249242_sup1 Supplementary information

## References

[JEB249242C1] Abbott, B. Y. B. C., Bigland, B. and Ritchie, J. M. (1952). The physiological cost of negative work. *J. Physiol.* 117, 380-390. 10.1113/jphysiol.1952.sp004755 14946742 PMC1392548

[JEB249242C2] Ackermann, M. A. and Kontrogianni-Konstantopoulos, A. (2011). Myosin binding protein-c: a regulator of actomyosin interaction in striated muscle. *J. Biomed. Biotechnol.* 2011, 636403. 10.1155/2011/636403 22028592 PMC3196898

[JEB249242C3] Ackermann, M. A., Kerr, J. P., King, B., Ward, C. W. and Kontrogianni-Konstantopoulos, A. (2015). The phosphorylation profile of myosin binding protein-c slow is dynamically regulated in slow-twitch muscles in health and disease. *Sci. Rep.* 5, 12637. 10.1038/srep12637 26285797 PMC4642540

[JEB249242C4] Barclay, C. J. and Curtin, N. A. (2023). Advances in understanding the energetics of muscle contraction. *J. Biomech.* 156, 111669. 10.1016/j.jbiomech.2023.111669 37302165

[JEB249242C5] Barclay, C. J., Woledge, R. C. and Curtin, N. A. (2007). Energy turnover for Ca^2+^ cycling in skeletal muscle. *J. Muscle Res. Cell Motil.* 28, 259-274. 10.1007/s10974-007-9116-7 17882515

[JEB249242C6] Barclay, C. J., Woledge, R. C. and Curtin, N. A. (2010). Inferring crossbridge properties from skeletal muscle energetics. *Prog. Biophys. Mol. Biol.* 102, 53-71. 10.1016/j.pbiomolbio.2009.10.003 19836411

[JEB249242C7] Baylor, S. M. and Hollingworth, S. (2007). Simulation of Ca2^+^ movements within the sarcomere of fast-twitch mouse fibers stimulated by action potentials. *J. Gen. Physiol.* 130, 283-302. 10.1085/jgp.200709827 17724162 PMC2151645

[JEB249242C8] Bhargava, L. J., Pandy, M. G. and Anderson, F. C. (2004). A phenomenological model for estimating metabolic energy consumption in muscle contraction. *J. Biomech.* 37, 81-88. 10.1016/S0021-9290(03)00239-2 14672571

[JEB249242C9] Burkholder, T. J. and Lieber, R. L. (2001). Sarcomere length operating range of vertebrate muscles during movement. *J. Exp. Biol.* 1536, 1529-1536. 10.1242/jeb.204.9.1529 11296141

[JEB249242C10] Curtin, N. A. and Barclay, C. J. (2023). The energetics of muscle contractions resembling *in vivo* performance. *J. Biomech.* 156, 111665. 10.1016/j.jbiomech.2023.111665 37327644

[JEB249242C11] Curtin, N. A., Gardner-Medwin, A. R. and Woledge, R. C. (1998). Predictions of the time course of force and power output by dogfish white muscle fibres during brief tetani. *J. Exp. Biol.* 201, 103-114. 10.1242/jeb.201.1.103 9390941

[JEB249242C12] Curtin, N. A., Woledge, R. C., West, T. G., Goodwin, D., Piercy, R. J. and Wilson, A. M. (2019). Energy turnover in mammalian skeletal muscle in contractions mimicking locomotion: effects of stimulus pattern on work, impulse and energetic cost and efficiency. *J. Exp. Biol.* 222, jeb203877. 10.1242/jeb.203877 31221738

[JEB249242C13] Ebashi, S., Endo, M. and Otsuki, I. (1969). Control of muscle contraction. *Q. Rev. Biophys.* 2, 351-384. 10.1017/S0033583500001190 4935801

[JEB249242C14] Farman, G. P., Tachampa, K., Mateja, R., Cazorla, O., Lacampagne, A. and De Tombe, P. P. (2008). Blebbistatin: Use as inhibitor of muscle contraction. *Pflugers Arch. Eur. J. Physiol.* 455, 995-1005. 10.1007/s00424-007-0375-3 17994251

[JEB249242C15] Fick, A. (1892). Neue beiträge zur kenntniss von der wärmeentwicklung im muskel. *Pflugers Arch. Eur. J. Physiol.* 51, 541-569. 10.1007/BF01663505

[JEB249242C16] Herzog, W. (2018). The multiple roles of titin in muscle contraction and force production. *Biophy. Rev.* 10, 1187-1199. 10.1007/s12551-017-0395-y PMC6082311 29353351

[JEB249242C17] Herzog, W. and Ait-Haddou, R. (2002). Considerations on muscle contraction. *J. Electromyogr. Kinesiol.* 12, 425-433. 10.1016/S1050-6411(02)00036-6 12435539

[JEB249242C18] Huxley, A. F. (1957). Muscle structure and theories of contraction. *Prog. Biophys. Biophys. Chem.* 7, 255-318. 10.1016/S0096-4174(18)30128-8 13485191

[JEB249242C19] Huxley, A. F. (2000a). Cross-bridge action: Present views, prospects, and unknowns. *J. Biomech.* 33, 1189-1195. 10.1016/S0021-9290(00)00060-9 10899327

[JEB249242C20] Huxley, A. F. (2000b). Mechanics and models of the myosin motor. *Philos. Trans. R. Soc. B Biol. Sci.* 355, 433-440. 10.1098/rstb.2000.0584 PMC1692758 10836496

[JEB249242C21] Kistemaker, D. A., Wong, J. D. and Gribble, P. L. (2010). The central nervous system does not minimize energy cost in arm movements. *J. Neurophysiol.* 104, 2985-2994. 10.1152/jn.00483.2010 20884757

[JEB249242C22] Koelewijn, A. D., Heinrich, D. and van den Bogert, A. J. (2019). Metabolic cost calculations of gait using musculoskeletal energy models, a comparison study. *PLoS ONE* 14, e0222037. 10.1371/journal.pone.0222037 31532796 PMC6750598

[JEB249242C23] Kovács, M., Tóth, J., Hetényi, C., Málnási-Csizmadia, A. and Seller, J. R. (2004). Mechanism of blebbistatin inhibition of myosin II. *J. Biol. Chem.* 279, 35557-35563. 10.1074/jbc.M405319200 15205456

[JEB249242C24] Lemaire, K. K., Baan, G. C., Jaspers, R. T. and van Soest, A. J. (2016). Comparison of the validity of Hill and Huxley muscle-tendon complex models using experimental data obtained from rat m. soleus *in situ*. *J. Exp. Biol.* 219, 977-987. 10.1242/jeb.144394 26896546

[JEB249242C25] Lemaire, K. K., Jaspers, R. T., Kistemaker, D. A., van Soest, A. J. and van der Laarse, W. J. (2019). Metabolic cost of activation and mechanical efficiency of mouse soleus muscle fiber bundles during repetitive concentric and eccentric contractions. *Front. Physiol.* 10, 760. 10.3389/fphys.2019.00760 31293438 PMC6599155

[JEB249242C26] Lichtwark, G. A. and Wilson, A. M. (2005). A modified Hill muscle model that predicts muscle power output and efficiency during sinusoidal length changes. *J. Exp. Biol.* 208, 2831-2843. 10.1242/jeb.01709 16043588

[JEB249242C27] Lichtwark, G. A. and Wilson, A. M. (2007). Is Achilles tendon compliance optimised for maximum muscle efficiency during locomotion? *J. Biomech.* 40, 1768-1775. 10.1016/j.jbiomech.2006.07.025 17101140

[JEB249242C28] Loiselle, D. S., Tran, K., Crampin, E. J. and Curtin, N. A. (2010). Why has reversal of the actin-myosin cross-bridge cycle not been observed experimentally? *J. Appl. Physiol.* 108, 1465-1471. 10.1152/japplphysiol.01198.2009 20133436

[JEB249242C29] Millard, M., Franklin, D. W. and Herzog, W. (2024). A three filament mechanistic model of musculotendon force and impedance. *eLife* 12, RP88344. 10.7554/eLife.88344 39254193 PMC11386956

[JEB249242C30] Miller, R. H., Umberger, B. R., Hamill, J. and Caldwell, G. E. (2012). Evaluation of the minimum energy hypothesis and other potential optimality criteria for human running. *Proc. R. Soc. B* 279, 1498-1505. 10.1098/rspb.2011.2015 PMC3282349 22072601

[JEB249242C31] Ranatunga, K. W. (2010). Force and power generating mechanism(s) in active muscle as revealed from temperature perturbation studies. *J. Physiol.* 588, 3657-3670. 10.1113/jphysiol.2010.194001 20660565 PMC2998218

[JEB249242C32] Rodman, P. S. and McHenry, H. M. (1980). Bioenergetics and the origin of hominid bipedalism. *Am. J. Phys. Anthropol.* 52, 103-106. 10.1002/ajpa.1330520113 6768300

[JEB249242C33] Sampaio de Oliveira, M. L. and Uchida, T. K. (2024). Phenomenological muscle constitutive model with actin–titin binding for simulating active stretching. *J. Biomech. Eng.* 147, 011002. 10.1115/1.4066564 39269663

[JEB249242C34] Selinger, J. C., O'Connor, S. M., Wong, J. D. and Donelan, J. M. (2015). Humans can continuously optimize energetic cost during walking. *Curr. Biol.* 25, 1-5. 10.1016/j.cub.2015.08.016 26365256

[JEB249242C35] Seow, K. N. and Seow, C. Y. (2022). Molecular events of the crossbridge cycle reflected in the force–velocity relationship of activated muscle. *Front. Physiol.* 13, 846284. 10.3389/fphys.2022.846284 35360243 PMC8960716

[JEB249242C36] Smith, N. P., Barclay, C. J. and Loiselle, D. S. (2005). The efficiency of muscle contraction. *Prog. Biophys. Mol. Biol.* 88, 1-58. 10.1016/j.pbiomolbio.2003.11.014 15561300

[JEB249242C37] Tran, K., Smith, N. P., Loiselle, D. S. and Crampin, E. J. (2009). A thermodynamic model of the cardiac sarcoplasmic/endoplasmic Ca2+ (SERCA) pump. *Biophys. J.* 96, 2029-2042. 10.1016/j.bpj.2008.11.045 19254563 PMC2717298

[JEB249242C38] Umberger, B. R. (2010). Stance and swing phase costs in human walking. *J. R. Soc. Interface* 7, 1329-1340. 10.1098/rsif.2010.0084 20356877 PMC2894890

[JEB249242C39] Umberger, B. R., Gerritsen, K. G. and Martin, P. E. (2003). A model of human muscle energy expenditure. *Comput. Methods Biomech. Biomed. Engin.* 6, 99-111. 10.1080/1025584031000091678 12745424

[JEB249242C40] van den Bogert, A. J., Hupperets, M., Schlarb, H. and Krabbe, B. (2012). Predictive musculoskeletal simulation using optimal control: Effects of added limb mass on energy cost and kinematics of walking and running. *Proc. Inst. Mech. Eng. Pt. P J. Sports Eng. Tech* 226, 123-133. 10.1177/1754337112440644

[JEB249242C41] van Soest, A. J. and Bobbert, M. (1993). The contribution of muscle properties in the control of explosive movements. *Biol. Cybern.* 204, 195-204. 10.1007/BF00198959 8373890

[JEB249242C42] van Soest, A. J., Casius, L. J. R. and Lemaire, K. K. (2019). Huxley-type cross-bridge models in largeish-scale musculoskeletal models; an evaluation of computational cost. *J. Biomech.* 83, 43-48. 10.1016/j.jbiomech.2018.11.021 30554816

[JEB249242C43] Zahalak, G. I. (1981). A distribution-moment approximation for kinetic theories of muscular contraction. *Math. Biosci.* 55, 89-114. 10.1016/0025-5564(81)90014-6 32835693

