# Supplementary material for: Validation of the energetics of a Huxley muscle–tendon complex model using experimental data obtained from mouse soleus muscle
Source: J Exp Biol. 2025 Aug 1;228(15):jeb249242. doi: 10.1242/jeb.249242 (PMC12377815; doi:10.1242/jeb.249242)
Supplement: Supplementary information [file jexbio-228-249242-s1.pdf]

## Supplementary Materials and Methods

In this appendix, we explore possible sources of the variance in the experimental data. Firstly, we note that, in line with our previous reporting (Lemaire et al., 2019) we observed a decreasing trend in mechanical and metabolic power over time within animals, in the 2 Hz, 0.25 mm concentric condition (Fig. S1, upper two panels). This was not associated with a clear trend in the mechanical efficiency, but the efficiency did substantially vary within these trials (Fig. S1, lower panel). From this we conclude that the variation in the data is not due to cell death and associated increased oxygen consumption, a finding that is supported by our previous reporting on the Succinate-Dehydrogenase stainings of these fibre bundles. We speculate that the systematic decline in mechanical and metabolic power output over time might be caused by an increased excitation threshold, whereas the stimulus amplitude remained the same during the course of the trial. We note that the stimulus amplitude in this experiment was limited by practical constraints, as too much current would lead to the formation of gas bubbles through electrolysis, that would interfere with the oxygen measurement.

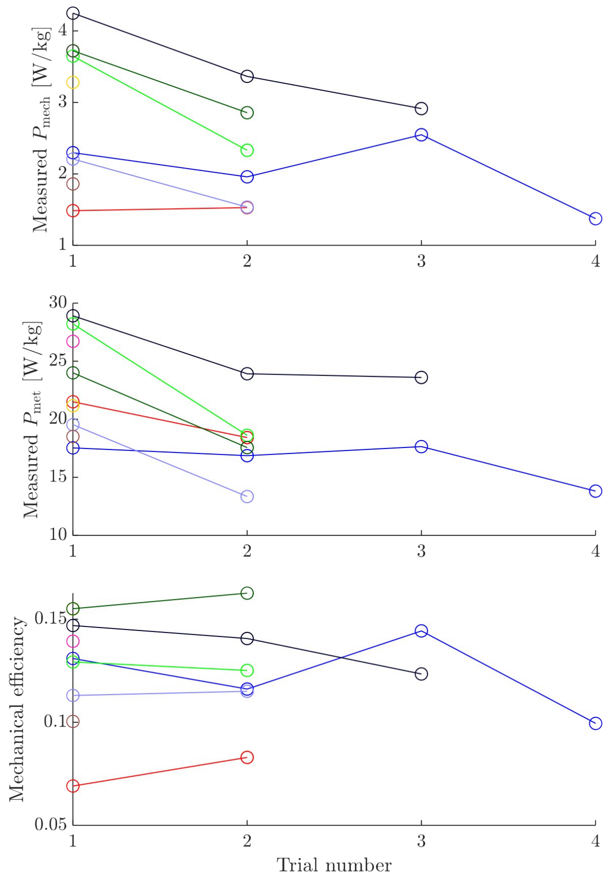


**Fig. S1. Measured, averaged metabolic power (A) and mechanical power (B) versus trial nr, for repeated trials of the 2 Hz, 0.25 mm concentric contraction condition.** Each dot represents data from one trial. Data pertaining to the same animal are connected by a line, animals are distinguished by color.

Journal of Experimental Biology • Supplementary information
